# The Humor of It

**Published:** 1887-03

**Authors:** Ad. Lippe

**Affiliations:** Philadelphia


### THE HUMOR OF IT.

AD. LIPPE, M. D., PHILADELPHIA.

This is the title of an editorial in the *Physicians' and Surgeons' Investigator*, Buffalo, N. Y., January, 1887. After reciting from a *Catalogue of Morbific Products, Nosodes, and other Remedies in High Potencies,* ostensibly published by Samuel Swan, M. D., New York, a large number of such nosodes, the editorial winds up with an attack on the International Hahnemannian Association. As a member of that Association, we give the attack as we find it, and shall make our individual comments on the humor the learned editors indulge in. They say:

"We would not occupy our space with this farrago of nonsense but for the humor which lies in the cover, and which is the imprimatur of the International Hahnemannian Society, with the motto, *Similia Similibus Curantur!* In the last volume of the *Transactions* of this Society is a paper from the author of this new pharmacopœa, and another from a writer in the course of which he makes the following apposite remarks: * * * 'Sectarians are you, who, knowing the fundamental character and constitution of Homœopathy, which comprehends and enlivens and renews the whole of the medical sciences, nevertheless wish to lower its law to the level of a rule of which you make use whenever it pleases you, mixing it with other hypotheticated laws that are affiliated with eclecticism, applying to it and changing it with those fossil proceedings of blind allopathy, whose darkness it has just come to put to flight, placing medicine among the exact sciences!'"

That a circular and catalogue under the above title is in existence is admitted. Whether Dr. Samuel Swan published it himself or whether it is published by some one else as a practical joke is an open question, and cannot be settled till Dr. Swan pleads guilty if he comes to be interrogated on his authorship. Till he pleads guilty or denies the authorship it behooves every charitable journal, as well as every fair-minded individual, to give him the benefit of the doubt. It is hardly to be believed that a sane man would publish such trash; it is hardly possible that a member of the I. H. A. would claim that *morbific matter will cure the diseases which produced it, if given in a high potency, even to the person from whom it was obtained.* And, pray, what has the I. H. A. to do with it at present? While the attention of the profession has been called to the existence of this catalogue, ostensibly published by Dr. Swan, a member of the American Institute and of the I. H. A., as well as of other medical societies, it is to be expected that such societies will fully investigate this circular, and it does not behoove the editors of journals to take a fling at any of the societies to which the ostensible author of this circular belongs.

The publication of this circular is a disgrace to the profession, but neither the author nor the Society to which he belongs should be held responsible till the scandal is fully investigated. The American Institute, as well as the I. H. A., meet but once a year, and it is but right to wait for their decisions after due investigation and after Samuel Swan, M. D., has been given due opportunity to "explain" his relation to the circular, and if he published it in reality, to show proof of the correctness of his assertions and propositions and also show that his methods are purely homœopathic, and as it appears on the surface purely isopathic, and as such antagonistic to Homœopathy, our opinion is that the editors of the above journal in their "*humor*" are rather "*previous*," that they seemingly have forgotten the old axiom that a man, as well as a society to which he belongs, should not be denounced "guilty" till such guilt is properly proven. People who live in glass houses must not throw stones.

